# Supplementary material for: Therapeutic deep brain stimulation disrupts movement-related subthalamic nucleus activity in parkinsonian mice
Source: eLife. 2022 Jul 4;11:e75253. doi: 10.7554/eLife.75253 (PMC9342952; doi:10.7554/eLife.75253)
Supplement: Supplementary file 1. [file elife-75253-supp1.docx]

**Supplementary Tables**

**Supplementary File 1a.**

| **Current Amplitude (µA)** | **Frequency (Hz)** | **Pulse Width (µs)** | **Frequency Category** |
| --- | --- | --- | --- |
| 200 | 5 | 60 | Low |
| 200 | 10 | 60 | Low |
| 200 | 15 | 60 | Low |
| 200 | 20 | 60 | Low |
| 200 | 40 | 60 | Low |
| 200 | 60 | 60 | Medium |
| 200 | 80 | 60 | Medium |
| 200 | 100 | 60 | Medium |
| 200 | 120 | 60 | High |
| 200 | 140 | 60 | High |
| 200 | 160 | 60 | High |
| 200 | 180 | 60 | High |

**Supplementary File 1b.**

| **Current Amplitude (µA)** | **Frequency (Hz)** | **Pulse Width (µs)** | **High vs Low Effect** |
| --- | --- | --- | --- |
| 200 | 60 | 60 | High |
| 200 | 60 | 100 | High |
| 200 | 80 | 60 | High |
| 200 | 100 | 60 | High |
| 200 | 120 | 60 | High |
| 200 | 140 | 60 | High |
| 200 | 160 | 60 | High |
| 225 | 80 | 80 | High |
| 175 | 20 | 50 | Low |
| 200 | 10 | 120 | Low |
| 200 | 120 | 20 | Low |
| 300 | 10 | 60 | Low |
| 400 | 1 | 120 | Low |

**Supplementary File 1c.**
